# Factors influencing decision-making power regarding reproductive health and rights among married women in Mettu rural district, south-west, Ethiopia

**DOI:** 10.1186/s12978-019-0813-7

**Published:** 2019-10-29

**Authors:** Afework Tadele, Amanuel Tesfay, Alemi Kebede

**Affiliations:** Population and family health, Jimma University, Jimma, Oromia Ethiopia

**Keywords:** Decision-making, Reproductive health and rights, Rural, Ethiopia

## Abstract

**Background:**

Women’s decision-making power regarding reproductive health and rights (RHR) was the central component to achieve reproductive well-being. Literatures agree that a women having higher domestic decision-making power regarding their health care were more likely to utilize health services. More than 80% of women in Ethiopia reside in rural areas where they considered as the subordinates of their husbands. This would restrict women to fully exercise their RHR. Thus, this study aims to determine the factors influencing the women’s decision-making power regarding RHR in Mettu rural district, South West Ethiopia.

**Methods:**

A community based cross-sectional study was done among 415 by using randomly selected married women of reproductive age from March to April 2017. Data was entered by using Epi-data manger 1.4 and analyzed by SPSS version 21. Descriptive and multivariate logistic regression analysis was carried out.

**Result:**

One hundred sixty-eight (41.5%) of the women had greater decision-making power regarding RHR. Woman’s primary education AOR 2.62[95% C. I 1.15, 5.97], secondary (9+) education AOR 3.18[95% C. I 1.16, 8.73] and husband’s primary education AOR 4.0[95% C. I 1.53, 10.42], secondary (9+) education AOR 3.95 [95% C. I 1.38, 11.26], being knowledgeable about RHR AOR 3.57 [95% C. I 1.58, 8.09], marriage duration of more than 10 years AOR 2.95 [95% C. I 1.19, 7.26], access to micro-credit enterprises AOR 4.26[95% C. I 2.06, 8.80], having gender equitable attitude AOR 6.38 [95% C. I 2.52, 12.45] and good qualities of spousal relation AOR 2.95 [95% C. I 1.30, 6.64] were positively influencing women’s decision-making power regarding RHR.

**Conclusion:**

More than four in ten rural women had greater decision-making power regarding RHR. External pressures (qualities of spousal relation, gender equitable attitude) and knowledge about RHR were found to influence women’s decision-making power. Public health interventions targeting women’s RHR should take into account strengthening rural micro-credit enterprises, qualities of spousal relations and priority should be given to women with no formal education of husband or herself and marriage duration of < 5 years.

## Plain English summary

This study examines multiple factors that can influence decision-making power of married women regarding reproductive health and rights (RHR). Through structured interviews of married women in the reproductive age, the study determined socio-demographic, socio-economic and reproductive health factors influence how much final say women have over the healthcare they receive and maintaining their reproductive health and rights. The study finds that less than half the women surveyed women possess greater decision-making power with regard to RHR. The factors that most influence this were found to be attending formal education of women and their husbands, having knowledge about RHR and gender equitable attitudes, accessing in micro-credit enterprises, quality of spousal relationships, and marital duration. We believe that our study makes a significant contribution to the literature, because it provides a multivariate analysis of the complex set of factors influencing women’s decision-making power has been studied in an isolated fashion thus far. This study is also significant as it provides cogent policy recommendations for the design and implementation of economic and non-economic interventions into women’s empowerment.

## Introduction

Women often have less power in relationships due to their economic, political and sociocultural status and may not be in a position to protect themselves from gender-based violence, and unwanted sexual intercourse, resulting in sexually transmitted infections and other sexual and reproductive health (SRH) problems [1]. This indicates that gender inequalities exacerbate a difference in SRH well-being and ill health, and sometimes life and death under condition of poverty [2]. Women constitute about two-thirds of the 1.4 billion people currently living in extreme poverty, and make up 60% of the 572 million working poor in the world [2].

In spite of the fact that remarkable improvement after the International Conference on Population and Development (ICPD), there was a resistant in an international fora regarding women’s reproductive health and rights (RHR) mainly due to the women’s social status and unwillingness to recognize women’s right to decide about their own RHR [3]. While all states promised to lower maternal mortality rates by 75% by 2015 as part of the Millennium Development Goals (MDGs), they have mostly failed to do so [4]. This was also largely due to gender inequalities [5] as decisions regarding reproductive choices, including where and when women ought to seek health care, usually made by their husbands [6]. Especially Married women adopt triple roles of production, reproduction and community management [6] i.e. the roles of wife, mother and caregiver as a primary identity for which they suffered disproportionately.

Studies conducted so far identified different factors like, age, educational status and income [7], occupation as being working women and non-working [8], marital status as polygamous marriages which were common among rural women and refusal of sexual intercourse resulting in their husbands turning to other wives [9] were found to be significantly influencing women’s decision making power regarding health-care services utilization.

According to a study conducted in Southern Ethiopia, 64.8% of married women in urban areas exercised decision-making power over contraceptive use, as against 43.1% of women in rural areas. Further within rural communities’ domestic decision-making power vested solely with the wife in only 14.71% of cases, while in 45.83% of cases, decisions were made solely by the husband [10]. This shows male dominance over women’s decision regarding sexual and reproductive health and rights (SRHR) especially in rural communities. Decisions regarding RHR i.e., the right of women to decide on their own health, whether or not, and how and when, to have children, and the power to influence and decide freely and responsibly on matters related to their sexuality go beyond merely services utilization. Increasing access to these rights is the long term solution to reproductive ill health through ensuring women’s reproductive wellbeing.

This study determined the major factors influencing women’s decision-making power regarding RHR, which are crucial for designing contextually appropriate and practically sound interventions to empower women on RHR. This study has also significant in influencing existing policy on a global agenda, and in realizing sustainable development goals which requires a multi-sectoral approach to be delivered at the rural level where community reside. Most of the factors that most influence the women’s decision-making power and maintaining rural women’s RHR requires multi-sectoral approach i.e. micro-credit enterprises from micro-economic and qualities of spousal relations from social workers including psychological interventions in building romantic life style for married rural women and men. Strengthening gender mainstreaming provide significant gain in realizing reproductive rights of rural women. Therefore, helping women to fully exercise their RHR is a great gain in improving the universal coverage of SRH.

## Materials and methods

### Study area and period

Mettu rural district is one of 13 districts in Illubabor zone, in the Oromia Regional state of Ethiopia. Mettu Rural District is located 600 km to the south-west of Addis Ababa, the capital city of Ethiopia. The study was conducted from March 14 to April 10, 2017.

#### Study design and population

A community based cross sectional study was employed, among randomly selected married women in reproductive age group residing in Mettu rural District at the time of the study and who had lived for at least for 6 months in the area and who had given birth at least once.

#### Sample size and sampling technique

Sample size was determined using Epi info™ Version 7.1.1, with the assumptions of confidence level = 95%, margin of error = 5% and power for double population proportion = 80%: P = proportion of married rural women who have decision making power to use family planning [3] **415** married women of reproductive age were sampled.

**Fig. 1:**
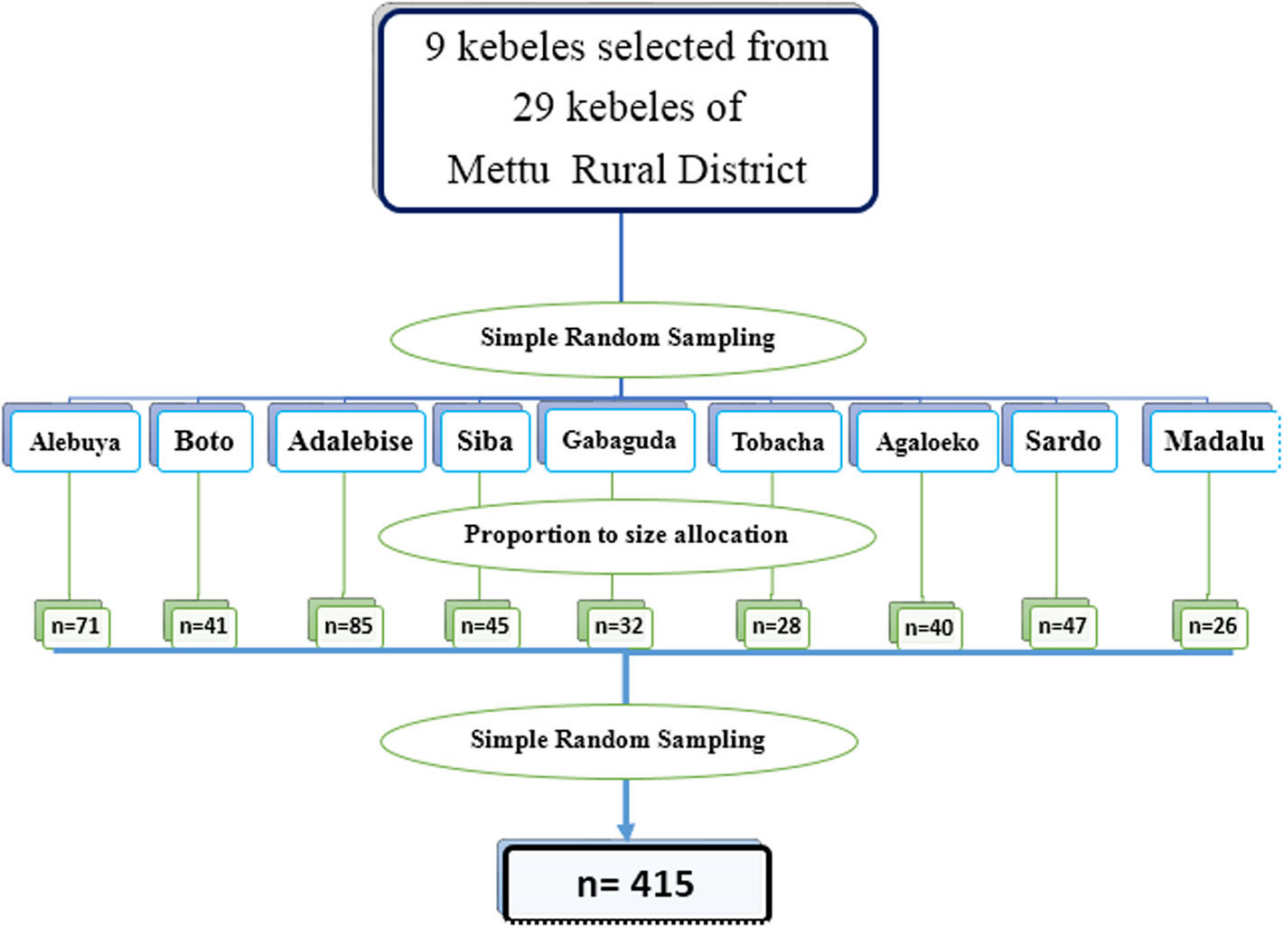
schematic presentation of sampling technique of the study Mettu district, 2017

Nine kebeles with a total population of 23,707 were selected from the 29 total kebeles, using Simple Random Sampling method. Following this, proportions of the sample were allocated to the selected nine kebeles, according to their size. In each of these kebeles, the total number of married women of reproductive age was obtained from the family folder of the community health information system available at the local health post and the recorded number was listed. The record number lists were then recoded in ascending order to create a frame, on which a table of randomized numbers was employed to identify study participants. The participants’ usual place of residence was then identified in collaboration with kebele leaders. Eligible married women of child-bearing age were interviewed in each kebele until the sample arrived at through SRS was covered. In cases of non-availability, such as where women were away from home, interviewers re-visited the household on at least three occasions before excluding a respondent for the reason of non-response see Fig. 1.

### Data collection instrument and quality management

Data were collected using a structured interviewer-administered questionnaire. The data collection instrument was developed in English after thoroughly revising related literatures and contextualized to suit to the research objective, local situations and translated to the local language (Afaan Oromo) and finally translated back to English for consistency. The questionnaires were administered in full privacy, and study subjects were interviewed about their reproductive history, socio-demographic profile, knowledge and gender role attitudes about RHR, decision-making power regarding SRHR and factors influencing their ability to make decisions. Before the actual data collection, the questionnaire was pre-tested on 5% of the sample size; then reliability test done yielding cronbach’s alfa > 0.7 taken for actual data collection. Training was given to data collectors & supervisors, with close supervision of the data collection process was carried out. The principal investigator and supervisors select for re-interview few married women in reproductive age group to check internal validity of the data.

### Measurement

Women’s decision-making power was measured using 11 questions based on pre-existing tools which were frequently used components of women’s decision-making power regarding RHR by various scholars [7–13] and adapted according to local contexts. The women were asked “Who in your family usually has the final say on the following decisions: 1. Health care for yourself, 2. when to become pregnant, 3. Number of children, 4. when to have sexual intercourse, 5. Place of birth of children, 6. Whether to use antenatal care services; 7. use of modern contraceptives, for each items the response was scored as follows: 2 if the woman was the sole decider, **1** if the decision involved someone else [husband/partner or someone else], and **0** otherwise; for non-users of, 8. Non user of Modern contraceptives and 9. Antenatal care; if their main reason for non-use was opposition from others (husband, mother in law, relative, religion etc.) a value of 0 was assigned and a **1** if otherwise. For decisions, 10. The right to assert on the use of male condoms and 11. the right to information on SRHR, the score was given as **1** for positive response and 0 for negative response.

Finally, a composite score on decision-making power on RHR was constructed and converted into a binary outcome based on a mean score developed: those scoring mean and above were categorized as higher decision-making power (coded as 1), whereas those scoring less than the mean were categorized as lower decision-making power (coded as 0).

**Women’s knowledge of RHR** was assessed by considering knowledge regarding the components of RHR that essentially address reproductive health and rights services such as modern contraception, safe child bearing, reproductive tract infections, sexually transmitted infections, HIV/AIDS, safe sexual behavior and key danger signs during labor and childbirth. The desired answers were coded as 1; while others were coded 0. A total 32 questions were asked to assess knowledge on RHR. Those mothers who scored above 70% (≥23) were categorized as knowledgeable, while those scoring less were categorized as less knowledgeable [14].

**Gender equitable attitudes** of married women were measured using 13 questions further constructed into a composite score. Each question had 5 Likert scale response options, based on the degree of agreement on statements about equity regarding RHR access and uptake. Based on the summative score, a score of above 80% of the distribution was categorized as having gender equitable attitudes, with lower scores categorized as having gender inequitable attitudes [10].

**The quality of spousal relationships:** was measured using five questions. Women scoring greater than the mean score were categorized as having good quality of spousal relationship, while those scoring less than the mean categorized as having poor quality of spousal relationship.

### Data processing and statistical analysis

The collected data were cleaned, edited, coded and entered into EpiData manager™ Version 4.1, and then exported to the Statistical Package for Social Sciences version 21.0. The data were examined for inconsistencies and missing values. To measure socio-economic status, a wealth Index was created using principal component analysis. After checking for multi-colinearities, complex structures, communalities and internal consistencies of the high loading items, composite scores were developed. An odds ratio with 95% confidence interval and *p*-value less than 0.05 was computed to assess the presence and degree of association and statistical significance between the dependent and independent variables. After checking for multi-collinearity to rule out the presence of linear association among explanatory variables, bivariate analysis was carried out for each predictor variable with the outcome variable to determine its independent effect. Variables that were found to be statistically significant at 0.25 in bivariate were entered into multivariate logistic regression model to develop the final model. The final results were presented in text and tables.

## Results

### Socio-demographic characteristics of the respondents

A total of 405 women were interviewed for the study, yielding a response rate of 97.6%. The mean (+ SD) age of the women was 29.13 (+ 6.53) years. As Table 1 shows, 81.2% of the women were Oromo by ethnicity. The religion of 35.8% respondents was protestant, followed by 31.1% orthodox and 28.9% Muslims. majority of the respondents (92.3%) were engaged in monogamous marriage. Nearly half (47.7%) of the participants had a family size of 3–4 with the average (+ SD) family size being 4.91(+ 1.74) see Table 1.

**Table 1:** Socio-demographic variables of married women in the reproductive age group in Mettu Rural District, South-West Ethiopia

Socio-demographic characteristics	Category	Frequency	Percent
Age group (in years)	15–19	10	2.5
	20–24	94	23.2
	25–29	116	28.6
	30–34	97	24.0
	35–39	56	13.8
	40–44	20	4.9
	45–49	12	3.0
Ethnicity	Oromo	329	81.2
	Amara	44	10.9
	Tigre	17	4.2
	Other^a^	15	3.7
Religion	Orthodox	126	31.1
	Muslim	117	28.9
	Protestant	145	35.8
	Others^b^	17	4.2
Educational status of respondent	no formal education	165	40.7
	primary (1–8)	166	41.0
	secondary and above	74	18.3
Family size	3–4	193	47.7
	5–6	145	35.8
	7 and above	67	16.5
	1- < 5 years	106	26.2
Duration of marriage	5 - < 10 years	95	23.5
	10 years and above	204	50.4

^a^ Sheka, Kefa, Gurage ^b^ Traditional (god), Wakeffata

### Married women’s decision making power on reproductive health and rights

**Fig. 2:**
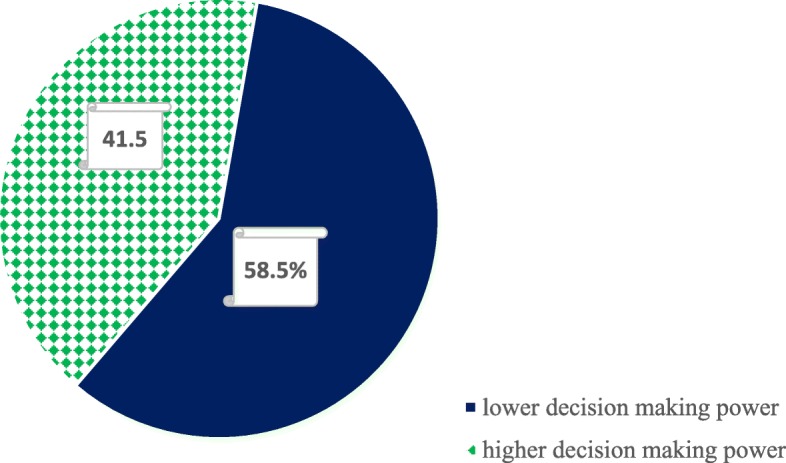
level of women’s decision making on RHR among married women of reproductive age group in Mettu Rural District, South-West Ethiopia March 14 to April 10, 2017

The study revealed that the mean (±SD) score for women’s decision making on reproductive health and rights was 7.50 (±2.64). Two hundred thirty-seven women (58.5%) had lower decision-making power on RHR while the remaining 168 (41.5%) had greater decision-making power on RHR see Fig. 2.

### Independent predictors of women’s greater decision-making power on reproductive health and rights

A bivariate analysis reveals that the formal education of respondents and their husbands, belonging in the middle and highest wealth tertiles, having gender equitable attitudes, being a member of micro-credit enterprises, having good quality of spousal relations, being knowledgeable about RHR, having a spousal age difference of less than 10 years and being married for longer than 5 years are all statistically significant for greater decision-making power on RHR.

After adjusting for potential confounders in the multivariate logistic regression shows that formal education (self and husband), being a member of micro-credit enterprises, having good quality of spousal relations, having a gender equitable attitude, being knowledgeable about RHR and having been married for ten or more years were found to be significant predictors of women’s greater domestic decision-making power on RHR.

A statistically significant difference was seen with regard to educational status, even after controlling for other variables. Women who had received primary education were two times more likely [AOR 2.62(95% C. I 1.15, 5.97)], and those who had received secondary (9+) or higher education were three times more likely [AOR 3.18(95% C. I 1.16, 8.73)] to have greater decision-making power than those who had no formal education. Further, when respondent’s husbands had received primary education they were four times more likely [AOR 4.00 (95% C. I 1.53, 10.42)], and if their husbands had received secondary (9+) or higher education, they were about four times more likely [AOR 3.95 (95% C. I 1.38, 11.26)], to have greater decision-making power than those whose husbands had no formal education.

Women who had access to micro-credit enterprises were four times more likely [AOR 4.26 (95% C. I 2.06, 8.80)] to have greater decision-making power as compared to those not had access to micro-credit enterprises. Similarly, those with gender equitable attitudes were six times more likely [AOR 6.38 (95% C. I 2.52, 12.45)] and those with good quality of spousal relations were about three times more likely (AOR 2.95 (95% C. I 1.30, 6.64)] to have greater decision-making power than their counterparts.

Respondents who had been married to their current husband for 10 or more years were about three times more likely [AOR 2.95 (95% C. I 1.19, 7.26)] as compared to less than 5 years of marital duration, and those who were more knowledgeable about RHR were three times more likely [AOR 3.57 (95% C. I 1.58, 8.09)] to have greater decision-making power than their counterparts see Table 2.

**Table 2:** Independent predictors of higher decision-making power on RHR in Mettu Rural District, South-West Ethiopia

Variables	Women’s decision making power on RHR			
	Higher *n* = 168 (%)	Lower *n* = 237(%)	COR[95% C.I]	AOR[95% C.I]
Educational status of respondent				
No formal education	28 (16.9)	138 (83.1)	1.0	1.0
Primary (1–8)	93 (58.9)	65 (41.1)	7.05 [4.21, 11.80] ^**^	2.62 [1.15, 5.97]^*^
Secondary(9+) and above	47 (58.0)	34 (42.0)	6.81 [3.74, 12.41] ^**^	3.18 [1.16, 8.73]^*^
Husband’s educational status				
No formal education	19 (17.0)	93 (83.0)	1.0	1.0
Primary (1–8)	73 (44.2)	92 (55.8)	3.88 [2.17,6.94] ^**^	4.00 [1.53, 10.42]^*^
Secondary(9+) and above	76 (59.4)	52 (40.6)	7.15 [3.90,13.12] ^**^	3.95 [1.38, 11.26]^*^
Wealth index				
Lowest tertile	31 (12.8)	105 (77.2)	1.0	1.0
Middle tertile	50 (37.3)	84 (62.7)	2.01 [1.18, 3.43] ^*^	1.04 [0.36, 2.99]
Highest tertile	87 (64.4)	48 (35.6)	6.13 [3.60, 10.46] ^**^	1.68 [0.56, 4.97]
Access to micro-credit enterprises				
Yes	96 (72.7)	36 (27.3)	4.61 [2.64, 8.06] ^**^	4.26 [2.06, 8.80]^**^
No	28 (25.9)	80 (74.1)	1.0	1.0
Gender equitable attitude				
Gender equitable	71 (77.2)	21 (22.8)	7.52 [4.37,12.95] ^**^	6.38 [2.52, 12.45]^**^
Gender inequitable	97 (31.0)	216 (69.0)	1.0	1.0
Knowledge about RHR				
Knowledgeable	77 (61.6)	48 (38.4)	3.33 [2.14,5.16] ^**^	3.57 [1.58, 8.09]^*^
Less knowledgeable	91 (32.5)	189 (67.5)	1.0	1.0
Quality of spousal relationships				
Good	120 (50.8)	116 (49.2)	2.60 [1.71,3.97] ^**^	2.95 [1.30, 6.64]^*^
Poor	48 (28.4)	121 (71.6)	1.0	1.0
Spousal age difference				
Less than 5 years	81 (54.7)	67 (45.3)	3.85 [2.14,6.94] ^**^	1.47 [0.48, 4.48]
5–9 years	66 (39.1)	103 (60.9)	2.04 [1.14,3.64] ^*^	1.22 [0.35,4.20]
10 years and above	21 (23.9)	67 (76.1)	1.0	1.0
Duration of Marriage				
1- < 5 years	25 (23.6)	84 (76.4)	1.0	1.0
5- < 10 years	36 (37.8)	59 (62.1)	1.97 [1.07,3.64] ^*^	1.23 [0.46, 3.31]
> 10 years	107 (52.5)	97 (47.5)	3.57 [2.11,6.04] ^**^	2.95 [1.19, 7.26]^*^

^*^Statistically significant at *p*-value < 0.05, ^**^ statistically significant at *p*-value < 0.001, *COR* crude odds ratio, *AOR* adjusted odds ratio, 1.0 reference category

## Discussion

Exercising reproductive health rights has been recognized as one of the pre-requisites for sustainable development in many developing countries including Ethiopia. Reproductive rights are among the human rights that should be protected. It is important for women to be able to take decisions regarding their own reproductive health and rights, especially during the reproductive period. When women possess greater decision-making powers in the household over their own reproductive health and rights, the health of the family as a whole is better protected, and thereby contributes to the productive forces of the country as a whole. In Ethiopia, a large majority of women reside in rural areas, where childbirth-related complications endanger maternal health outcomes. The need to prevent these complications has been emphasized in the slogan “No mother should die to give life.” Empowering women with regard to RHR plays a large role in tackling these issues. Women’s decision-making alone or jointly with their husbands is one of factors that contributes to effective and sustained use of RHR services, thereby resulting in early prevention of maternal mortality.

In the current study, only 168 (41.5%) of a total of 405 married women in the reproductive age group had greater decision-making power on reproductive health and rights. This is comparable to the findings of a cross-sectional study conducted in Bale Zone in 2014, which showed that only 39.5% of women had greater decision-making power regarding maternal and child health care [15]. The current findings are also comparable to those from a cross-sectional study conducted in Southern Ethiopia in 2011, which showed that 43.1% of married rural women had decision-making power regarding modern contraceptive use [10].

The findings of the current study were lower than those of an Ethiopian national-level study, which showed that 71.6% of rural women participated in decisions regarding their own health care [13]. This could be due to the additional components of RHR considered as composite variables in the current study.

This study found multiple predictors of the level of domestic decision-making power on RHR of married rural women in the reproductive age group level. It demonstrated that women who had been in a marital union for ten or more years were more likely to have greater decision-making power on RHR than those who had been in a marital union for less than 5 years. This finding is consistent with a study from Nekemte in West Ethiopia, which reported that women who had been married for less than 5 years were less likely to be the household decision-maker than those who had been in a marital union for ten or more years [16].

In the present study, women having access to micro-credit enterprises were more likely to have greater decision-making power. Similarly, a qualitative study in Rural Tanzania in 2012, supports the finding that microcredit, by itself, can significantly contribute to non-economic aspects of empowerment, regardless of whether one has taken a loan or not [17]. Another cross sectional study in Kenya in 2013 showed a positive relationship between women’s access to credit and reproductive decision-making roles [18]. This is because poverty at different levels, i.e., country, household, and so on, can have a severe impact on women and increases their economic dependency. Microfinance has a positive impact on women’s income [19]. Access to microcredit can facilitate both economic and non-economic improvements in rural women’s lives. Changes in basic material possession are expected to generate increased self-esteem, respect, and other forms of empowerment for women beneficiaries.

The current study also demonstrates that knowledge regarding RHR and gender equitable attitudes are associated with greater decision-making power. Studies in southern Ethiopia and Bale Zone similarly showed that knowledge and gender equitable attitudes were associated with higher levels of women’s decision-making power [12, 15].

Two other factors found to be positively associated with women’s decision-making power in this study were exposure to formal education of women and their husbands. This finding was supported by a systematic review of literature on women’s autonomy in health care decision-making in developing countries, conducted in 2016, which reported that highly educated women are more likely to be knowledgeable about their own health, have more self-confidence, and be more assertive than those with less or no education [7]. This might be due to men’s awareness of his duties and responsibilities as husband and head of the family and also makes women could easily influence their husbands and significant others.

Quality of spousal relations was also found to be a predictor of women’s greater decision-making power on RHR in the present study. Women having good spousal relations are three times more likely to have greater decision-making power. This shows that good spousal relations enhance communication, which generates trust and open discussions among spouses, and mutual respect of different perspectives regarding mandatory decisions.

Although there is no commonly agreed-upon definition for decision-making power on reproductive health and rights, and the complexity of its measurement, this study addresses the most frequently used components of women’s decision-making power regarding RHR proposes by various scholars [7–10, 12, 13]. Social desirability and recall bias may compromise the findings, since many gender sensitive responses may have been masked over, despite the conduct of interviews in conditions of privacy, and despite the use of multiple questions to minimize these problems. This study excludes the responses of men, and only relies on those of women. However, there is no guarantee that women’s responses alone will definitely reveal problems related to women’s reproductive health and rights.

## Conclusion

Less than half of the respondents have higher decision-making power on RHR in their households. Married women will not fully exercise their reproductive health and rights equally with their husband due to numerous challenges. Having husband with formal educational status, own formal education, being knowledgeable about RHR, having gender equitable attitude, good quality spousal relationship, being a member of micro-credit enterprises and ten and above year marital union were the independent predictors of married women of childbearing age higher decision-making power on RHR. Encouraging qualities of spousal relations among married rural women with strategies that strengthen partner communication and assertiveness skills to improve RHR decision making process and Expanding micro-credit enterprises to rural areas with promotion of women membership recommended. Further qualitative studies on longitudinal base to explore the sociocultural factors like taboos, values, believes on sexuality etc. were recommended for women’s higher decision making power on RHR in their households.

## Data Availability

The data sets used and/or analyzed during the current study are available from the corresponding author on reasonable request.

## Abbreviations

AOR: Adjusted Odds Ratio
ICPD: International Conference on Population and Development
MDG: Millennium Development Goals
RHR: Reproductive Health and Rights
SPSS: Statistical Package for Social Sciences
SRH: Sexual and Reproductive Health
SRHR: Regarding sexual and reproductive health and rights
